# Large-conductance calcium-activated potassium channel haploinsufficiency leads to sensory deficits in the visual system: a case report

**DOI:** 10.1186/s13256-022-03387-7

**Published:** 2022-05-05

**Authors:** Olivier Perche, Fabien Lesne, Alain Patat, Susanne Raab, Roy Twyman, Robert H. Ring, Sylvain Briault

**Affiliations:** 1 Genetic Department, Centre Hospitalier Régional d’Orléans, Orléans, France; 2 UMR7355, Immunologie et Neurogénétique Expérimentales et Moléculaires (INEM), Centre National de la Recherche Scientifique (CNRS), Orléans, France; 3 Experimental and Molecular Immunology and Neurogenetics, University of Orléans, Orléans, France; 4 Kaerus Bioscience Ltd, London, EC1Y 4YX UK; 5 Department of Pharmacology and Physiology, Drexel University College of Medicine, Philadelphia, PA USA; 6 Amron Neuroscience, LLC, Darby, MT USA

**Keywords:** BKCa, *KCNMA1*, Electroretinography, Contrast sensitivity, Case report

## Abstract

**Background:**

Mutations in the genes encoding the large-conductance calcium-activated potassium channel, especially *KCNMA1* encoding its α-subunit, have been linked to several neurological features, including intellectual disability or autism. Associated with neurodevelopmental phenotypes, sensory function disturbances are considered to be important clinical features contributing to a variety of behavioral impairments. Large-conductance calcium-activated potassium channels are important in regulating neurotransmission in sensory circuits, including visual pathways. Deficits in visual function can contribute substantially to poor quality of life, while therapeutic approaches aimed at addressing such visual deficits represent opportunities to improve neurocognitive and neurobehavioral outcomes.

**Case presentation:**

We describe the case of a 25-year-old Caucasian male with autism spectrum disorder and severe intellectual disability presenting large-conductance calcium-activated potassium channel haploinsufficiency due to a de novo balanced translocation (46, XY, t [9; 10] [q23;q22]) disrupting the *KCNMA1* gene. The visual processing pathway of the subject was evaluated using both electroretinography and visual contrast sensitivity, indicating that both retinal bipolar cell function and contrast discrimination performance were reduced by approximately 60% compared with normative control values. These findings imply a direct link between *KCNMA1* gene disruption and visual dysfunction in humans. In addition, the subject reported photophobia but did not exhibit strabismus, nystagmus, or other visual findings on physical examination.

**Conclusions:**

This case study of a subject with large-conductance calcium-activated potassium channel haploinsufficiency and photophobia revealed a visual pathway deficit at least at the retinal level, with diminished retinal light capture likely due to bipolar cell dysfunction and an associated loss of contrast sensitivity. The data suggest that large-conductance calcium-activated potassium channels play an important role in the normal functioning of the visual pathway in humans, and that their disruption may play a role in visual and other sensory system symptomatology in large-conductance calcium-activated potassium channelopathies or conditions where disruption of large-conductance calcium-activated potassium channel function is a relevant feature of the pathophysiology, such as fragile X syndrome. This work suggests that the combined use of physiological (electroretinography) and functional (contrast sensitivity) approaches may have utility as a biomarker strategy for identifying and characterizing visual processing deficits in individuals with large-conductance calcium-activated potassium channelopathy.

*Trial registration* ID-RCB number 2019-A01015-52, registered 17/05/2019.

## Background

The large-conductance calcium (Ca^2+^)-activated potassium channel (BKCa, MaxiK, KCa1.1, *slo*1) is a unique member of the K channel family that is characterized by a large single-channel conductance that can be activated by both membrane depolarization (voltage) and elevation of intracellular free calcium [1, 2]. The BKCa channel comprises a homotetramer of pore-forming α-subunits encoded by the *KCNMA1* gene that are localized to the plasma membrane. The structure of each pore-forming α-subunit comprises seven membrane-spanning segments (S0–S6) that include a voltage-sensing domain (S0–S4), a pore-gate domain (S5-P-S6), and a cytosolic C-terminal domain involved in Ca^2+^ sensitivity [1, 2]. BKCa channel activity is involved in the integration of various cellular and molecular signaling events via modulation of membrane excitability and Ca^2+^ homeostasis, including modulating neurovascular coupling and regulating vascular, respiratory tone, and especially neurotransmitter release, since BKCa channels are located at axon terminals and dendrites [1, 2].

*KCNMA1* mutations have increasingly been linked to several neurological disorders, including epilepsy, cerebellar ataxia, paroxysmal movement disorders, intellectual disability (ID), or autism, underscoring the important role of BKCa channels in controlling neuronal excitability [1, 2]. Since 2005, more than 20 different mutations of *KCNMA1* have been reported, with a spectrum of clinically defined pathological and behavioral phenotypes collectively referred to as “*KCNMA1*-linked channelopathy” [1–3]. This mutational spectrum encompasses both gain-of-function and loss-of-function alteration of BKCa channel activity, as well as several variants of unknown significance [1, 4]. Interestingly, an indirect BKCa channelopathy has been described in the literature, presenting with an ID phenotype associated with a nonsense mutation (R419X) in the *CRBN* gene, which encodes a cytosolic protein (CRBN, cereblon) that promotes the cell surface expression of BKCa channels in neurons [5, 6]. In addition, decreased protein expression of *KCNMA1* resulting in reduced BKCa channel activity has also been observed in fragile X syndrome (FXS) [7]. Moreover, a novel missense mutation c.413G > A (R138Q) in the fragile X mental retardation 1 locus (*FMR1*) leads to an FXS-like phenotype with a principal hallmark of reduced BKCa activity [8].

Genetic inactivation of the BKCa α-subunit in murine models leads to neurodevelopmental disorders characterized by the absence of experience-dependent increase in prepulse inhibition in electrophysiological measurements and slowed spatial learning in the Morris water maze [9]. Taken together, the human observations and animal findings suggest that BKCa channels are key contributors to neurodevelopment and normal neuronal communication.

BKCa channels play an important role in the first, neuronal signaling stage of visual processing. BKCa currents are expressed in key retinal cell types, and genetic knockout of the BKCa channel in mice (*Kcnma1*^−/−^) revealed impairments in the visual retinal pathway with a hallmark of reduced retinal response to light [10]. These findings, along with the observation of disturbances in sensory processing, are a prominent feature of neurodevelopmental disorders [11], prompting us to investigate the visual processing pathway in a subject with *KCNMA1* haploinsufficiency using electroretinography (ERG) and visual contrast sensitivity (CS). These novel and complementary biomarkers of sensory deficits in the visual system have recently been shown to be practical for assaying visual system function in subjects presenting neurobehavioral phenotypes and have excellent translational utility between a genetic mouse model (*Fmr1*^*−/y*^) of FXS and human FXS subjects [12].

## Case presentation

### Clinical symptoms and findings

The first clinical description of the current patient, a nonconsanguineous *KCNMA1* haploinsufficiency male (Caucasian), was when he was 6 years old [4]. His clinical presentation was associated with autistic features and severe ID that consisted of impairments in reciprocal social interactions and communication skills, lack of spoken language, poor communicating gestures, and restricted, stereotyped behaviors. Results of the Psychoeducational Profile–Revised indicated a developmental score equivalent to an age of 23 months and confirmed the associated diagnosis of severe intellectual disability according to DSM–IV criteria. Moreover, a diagnosis of autistic disorder was confirmed by administration of the Autism Diagnostic Interview–Revised and the Childhood Autism Rating Scale. Physical examination revealed normal growth parameters, and there were no discernible dysmorphic features. Electroencephalogram and cranial magnetic resonance imaging findings were normal. The patient’s parents were nonconsanguineous, and family history lacked confounding medical or neuropsychiatric disorders.

Recently, at 25 years of age, the subject still presented the same clinical features but additionally exhibited perseverative ideas, soliloquy (playing several roles with voice modification), repetitive speech, hyperactivity and aggressiveness, and outbursts of anger. Evidence of sensory abnormalities were present, including atypical reactions to odors (e.g., hypersensitivity to food odor), tactile defensiveness, and persistent (daily) sensation-seeking, especially auditory self-stimulation. Caregivers specifically reported disturbances in ocular and visual systems, with the subject exhibiting pronounced sensitivity to light and evidence of photophobia (e.g., avoiding going out on sunny days, hiding his eyes with his hands, and closing the shutters all day long). There were no notable findings upon physical examination of the eyes, including absence of nystagmus and strabismus. His stature was within normal range for age, but with moderate obesity and body mass index of 32.7 kg/m^2^. Written informed consent was obtained from the patient’s caregiver.

### Genetic findings

In a previous investigation, karyotyping of the subject and his parents revealed a de novo balanced translocation (46, XY, t [9; 10] [q23;q22]) [4]. In addition, although no gene was observed in 9q23 region, the 10q22 region breakpoint was localized in the *KCNMA1* gene (NCBI NM_002227, MIM number [*600150]), encoding for the BKCa channel [4]. More recently, transcriptomic analysis performed on lymphoblastoid cell line of the patient showed haploinsufficiency of the gene (*KCNMA1*^+/–^) characterized by 50% decreased *KCNMA1* mRNA expression (data not shown), which is in accordance with the 50% decreased mRNA expression and BKCa activity measured using conventional patch-clamp electrophysiology originally reported by Laumonnier and collaborators [4]. Sequencing confirmed the localization of the breakpoint in the first intron of the *KCNMA1* gene, with a microdeletion of 14 pb [4]. No other significant mutations were observed. CGG repeat detection was normal (32 ± 1 CGG), and no mutation was detected in the *FMR1* gene (Xq27.3) by exome sequencing (data not shown), thus the subject did not express an FXS genotype. In addition, transcriptomic arrays on lymphoblastoid cells showed that *KCNMA1* mRNA was decreased by 50%, as also demonstrated by Laumonnier *et al.* in 2006, and that *FMR1* mRNA was expressed at wild-type level (data not shown).

### Electroretinography and contrast sensitivity (ERG-CS) findings

Combined ERG and CS using methods found to be feasible and reliable in population presenting neurobehavioral phenotypes such as FXS was performed with this subject, as described previously [12]. Electroretinograms (ERGs) were recorded by using a RETeval system (LKC Systems), a FDA 510(k) cleared, non-mydriatic full-field ERG device commonly used clinically in ophthalmic settings. ERGs were recorded in light-adapted (LA) conditions in accordance with standards established by the International Society of Clinical Electrophysiology of Vision (ISCEV) [13]. ERG could not be performed adequately in a dark-adapted environment due to the subject’s anxiety in the dark. CS was assessed using the LEA SYMBOLS^®^ low-contrast test, as described previously [12].

**Fig. 1:**
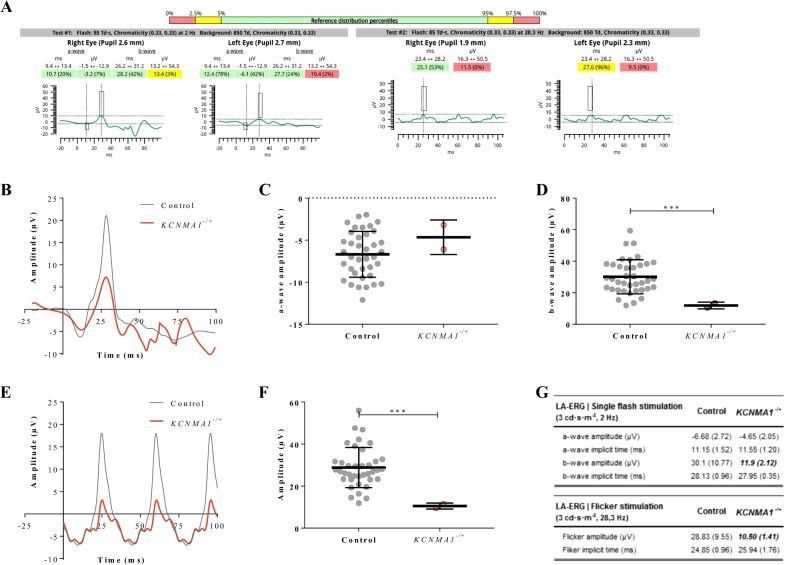
Summary data from ERG single flash and flicker stimulation. **A** Example RETeval report, showing measurements for the ISCEV standard LA single flash and flicker protocol. **B** ERG waveform trace summarizing the comparison of ERG recordings from both the *KCNMA1*^*−/+*^ subject (red) and a published healthy control (black) cohort [12] in response to stimulation with the ISCEV standard LA single flash protocol. Summary of LA-ERG (**C**) a-wave and **D** b-wave amplitude recordings measured in response to stimulation with single flash light protocol. **E** Representative raw waveform trace of LA-ERGs produced by flicker light stimulation protocol from the *KCNMA1*^*−/+*^ subject (red) and data from a published healthy control cohort of similar age [12] (black) in response to a 28.3 Hz train of repeated flashes of light (flickers protocol). **F** Comparison of LA-ERG waveform parameters recorded from the *KCNMA1*^*−/+*^ subject and the published healthy control cohorts in response to 28.3 Hz flicker stimulation [12]. **G** Summary data table for LA-ERG single flash and flicker protocol

Results from the RETeval device for this subject showed some ERG waveform measures to be out of the normal range compared with the RETeval normative database [14, 15] in both ISCEV standard LA single flash (3 cd s m−2, 2 Hz) and ISCEV standard LA flicker stimulation (3 cd s m−2, 28.3 Hz) stimulations. For both eyes, the LA single flash b-wave amplitude was below the 3% percentile of the reference distribution (3% and 2%, respectively), and the retinal response to the LA flicker stimulation was smaller than all references value at a 0% percentile of the reference distribution [14, 15] (Fig. 1A).

Similar differences in the subject’s ERG are evident when current results are compared with normative reference values of a separate age-similar cohort of healthy volunteers [12]. Indeed, ISCEV standard LA single flash (3 cd s m−2, 2 Hz) analysis revealed that the mean b-wave amplitude of the *KCNMA1*^*+/–*^ subject was significantly decreased (*p* = 0.0051) by 60.4% compared with healthy volunteers (control group, Fig. 1A, C, F), whereas no significant alterations of the a-wave were observed (Figure 1B, C, G). ISCEV standard LA flicker stimulation (3 cd s m−2, 28.3 Hz) revealed a significant impairment as shown by a 63.6% decreased amplitude (*p* = 0.0026) in the *KCNMA1*^*+/−*^ subject compared with a healthy volunteer (control group, Fig. 1E–G). No differences in implicit time were observed for single flash a- or b-waves, or in the flicker response (Fig. 1G).

**Fig. 2:**
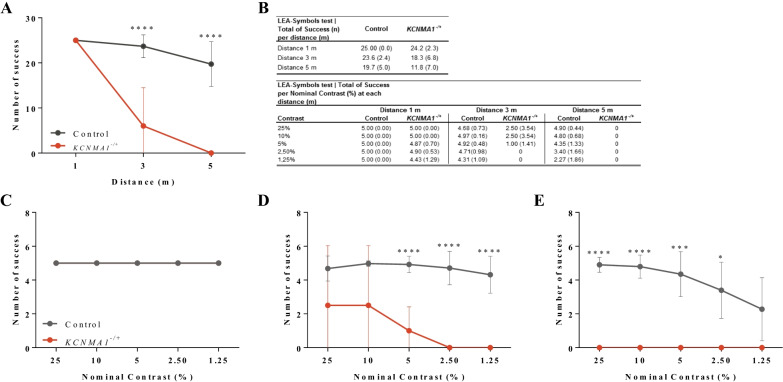
Data summary from LEA SYMBOLS low-contrast sensitivity test. **A** When calculating the total number of successes to discriminate symbols (25 maximum) for each of the three viewing distances (1, 3, and 5 m), significant reductions in scores from the *KCNMA1*^*−/+*^ subject were observed at 3 m and 5 m. **B** Summary data table for LEA SYMBOLS low-contrast sensitivity test. **C** Although no difference was observed at 1 m of distance, **D** the *KCNMA1*^*−/+*^ subject exhibited significantly lower contrast sensitivity compared with published data for healthy volunteers [12] at nominal contrast values of < 5% for 3 m of distance and **E** no response at all at 5 m of distance

In CS testing, statistical analysis of LEA SYMBOLS data from the *KCNMA1*^*+/–*^ subject revealed significant decreases in total success scores (sum of success to discriminate the symbols at three distances) when compared with data from a published age-similar healthy volunteer cohort of similar age (Fig. 2A, B) [12]. Deficits in CS were specifically present at the 3 m (*p* < 0.0001) and 5 m (*p* < 0.0001) testing distances (Fig. 1A, B). Regarding nominal contrast, no difference was observed at the 1 m distance at any nominal contrast level between the *KCNMA1*^*+/-*^ subject and the published healthy volunteer group (Fig. 1C). At the 3 m distance, the performance of the *KCNMA1*^*+/–*^ subject was significantly (*p* < 0.0001) lower compared with the published healthy volunteer group, beginning at the 5% level of nominal contrast and increasingly worse for lower nominal contrasts (2.5% and 1.25%) (Fig. 1D). At the 5 m distance, the *KCNMA1*^*+/–*^ subject failed to discriminate contrasts (Fig. 1E).

## Discussion and conclusions

BKCa channels are expressed in a variety of retinal cells such as rod and cone photoreceptors [16], bipolar cells [17], amacrine cells [18], and ganglion cells [19]. This expression pattern suggests a pivotal role of BKCa channels in retinal function and light perception as highlighted in *Kcnma1*^*−/−*^ mice, where the absence of BKCa leads to a significant reduction of ERG signals at high scotopic and low mesopic stimulus intensities [10]. Indeed, ERGs in *Kcnma1*^*−/−*^ mice are characterized by a reduced b-wave amplitude in dark-adapted conditions for a nocturnal animal without modification of either the a- or b-wave implicit time [10]. This phenotype is consistent with the role of BKCa in retinal neurons since BKCa channels control glutamate release at the photoreceptor synapse [16] and regulate the flow of excitatory synaptic transmission from the photoreceptor pathway to the ganglion cells via the amacrine/bipolar cell pathway [20]. Thus, BKCa channels are involved in signaling of light capture and downstream regulation of neurotransmission of light signals.

Interestingly, investigation of ERG and CS in the *Kcnma1*^*−/−*^ mouse model yielded similar findings to those observed in this *KCNMA1*^*−/+*^ subject. The substantial reduction in ERG b-wave amplitude (60.4% decrease) shows a bipolar cell signaling deficit in this subject as these cells are mainly involved in b-wave genesis. LA flicker stimulation also resulted in a substantial (63.9%) decrease in flicker wave amplitude, implying sensitivity of the retinal signaling to rapidly changing stimuli that emphasize the processing and communication pathways that lead out of the retina. Although background light conditions were different in the clinical investigation (light-adapted) versus the experimental conditions in *Kcnma1*^*−/−*^ mice (dark-adapted), the commonality of the bipolar cell dysfunction is supported by the decreased flicker response in the *KCNMA1*^*−/+*^ subject since this response is dominated by post-light-receptor circuit elements, particularly the ON and OFF bipolar cells that interact to shape the steady-state flicker ERG response [21]. Overall, the retinal electrophysiology abnormalities observed in the *KCNMA1*^*−/+*^ subject are similar to those observed in *Kcnma1*^*−/−*^ mice. BKCa channels are pivotal for normal regulation of retinal neurotransmission and thus visual pathway function, as proposed in murine models [10, 16, 20].

Using both ERG and CS, we have established that electrophysiologic abnormalities in the *KCNMA1*^*-/+*^ subject presented here are associated with a functional visual impairment that further implicates a role for BKCa channels in visual perception in humans. The subject exhibited a higher contrast sensitivity threshold and lower performance (55.7% decreased) to discriminate several contrast intensities starting at a distance of 3 m or greater. CS changes have direct behavioral consequences on an individual’s interaction and perception of their environment since it is defined by the threshold between the visible and invisible, and thus the ability to detect subtle differences in shading, patterns, in detecting objects without clear outlines, and discriminating objects or details from their background [12]. Interestingly, this *KCNMA1*^*−/+*^ subject’s caregiver reported that the subject exhibited avoidance behavior in front of a bright light source or during sunny days. The subject avoids bright light by staying inside the home with the curtains closed or wears sunglasses at home, suggesting a photophobia phenotype. Photophobia can be defined as a reduction of contrast sensitivity caused by glare light and thus a general aversion to light [22–24]. A link between contrast discrimination impairments and photophobia phenotypes has been described previously and suggested to have origins in retinal cell function, especially in cone-driven retinal pathways [22–25]. Interestingly, the specific b-wave ERG response abnormalities in this case also suggest involvement of cone-driven retinal pathways.

BKCa impairments have been described in FXS [7] as well as in the *Fmr1*^*−*/y^ genetic mouse model of FXS. In both *Fmr1*^*−*/y^ mice and FXS patients, genetic loss of FMRP leads to a reduction of BKCa α-subunit protein expression [7, 26–28]. In the murine *Fmr1*^*−*/y^ model, protein expression of BKCa α-subunit is reduced by as much as 50%, resulting in a corresponding 50% decrease in channel activity [7].

All these data mimic the BKCa haploinsufficiency phenotype found in our *KCNMA1*^*−/+*^ subject. It is important to note that modulating the BKCa activity with small-molecule channel openers has been shown to rescue a broad spectrum of behavioral phenotypes in the *Fmr1*^*−/y*^ mouse model of FXS [7, 28, 29]. Interestingly, using the combination of ERG and CS (ERG-CS) in a FXS cohort, we recently identified visual system electrophysiological and functional impairments characterized by decreased ERG b-wave and flicker wave amplitudes and lower CS discrimination ability [12]. The data obtained from that same use of the ERG-CS biomarker strategy in this *KCNMA1*^*−/+*^ subject are remarkably similar to those obtained in the FXS patients. Therefore, based on the BKCa expression pattern and role in the mammalian retina and the abnormalities of FMRP expression in retina, we suggest that part of the ERG-CS phenotype of the FXS population is due to a functional BKCa deficit linked to loss of FMRP in retina. In other words, BKCa impairments observed in FXS should be the main origin of the ERG-CS deficits, reinforcing the role of the BKCa channel in the FXS phenotype.

In summary, our study shows that BKCa haploinsufficiency leads to a visual pathway deficit associated with a light-capturing deficit at the retinal level, and with a functional alteration in contrast sensitivity associated with photophobia symptoms. The data from this case report suggest that BKCa channels play a pivotal role in visual pathway physiology. Interestingly, visual pathway data from the *Fmr1*^-/y^ murine model of FXS [7] and human FXS patients [12], both implicating BKCa channel dysfunction, suggest that BKCa channels play a major role in the functional and behavioral visual pathway impairment in the FXS phenotype. Further studies would be helpful to confirm these findings in broader FXS and *KCNMA1*-linked channelopathy populations such as Liang–Wang syndrome (LIWAS) (OMIM: 618729). Finally, ERG-CS appears to be good complementary biomarker to identify and characterize visual processing deficits in BKCa channelopathies and has potential utility in the development of therapeutics for conditions involving BKCa dysfunction.

## Data Availability

The data that support the findings of this study are available from Dr. Robert H. Ring (Kaerus Bioscience Ltd CEO), but restrictions apply to the availability of these data, which were used under license for the current study and so are not publicly available. Data are however available from the authors upon reasonable request and with permission of Robert H. Ring.

## Abbreviations

CS: Contrast sensitivity
ERG: Electroretinography
ID: Intellectual disability
LA: Light-adapted
ISCEV: International Society of Clinical Electrophysiology of Vision
